# Root-specific expression of *CsNPF2.3* is involved in modulating fluoride accumulation in tea plant (*Camellia sinensis*)

**DOI:** 10.1093/hr/uhaf072

**Published:** 2025-03-03

**Authors:** Huiliang Niu, Junjie Wang, Zhiwei Liao, Yangjuan Deng, Qi Chen, Chuanyi Peng, Guijie Chen, Ruyan Hou, Xiaochun Wan, Zhaoliang Zhang, Huimei Cai

**Affiliations:** National Key Laboratory for Tea Plant Germplasm Innovation and Resource Utilization, School of Tea & Food Science and Technology, Anhui Agricultural University, 130 West Changjiang Road, Hefei, Anhui 230036, China; National Key Laboratory for Tea Plant Germplasm Innovation and Resource Utilization, School of Tea & Food Science and Technology, Anhui Agricultural University, 130 West Changjiang Road, Hefei, Anhui 230036, China; National Key Laboratory for Tea Plant Germplasm Innovation and Resource Utilization, School of Tea & Food Science and Technology, Anhui Agricultural University, 130 West Changjiang Road, Hefei, Anhui 230036, China; National Key Laboratory for Tea Plant Germplasm Innovation and Resource Utilization, School of Tea & Food Science and Technology, Anhui Agricultural University, 130 West Changjiang Road, Hefei, Anhui 230036, China; National Key Laboratory for Tea Plant Germplasm Innovation and Resource Utilization, School of Tea & Food Science and Technology, Anhui Agricultural University, 130 West Changjiang Road, Hefei, Anhui 230036, China; National Key Laboratory for Tea Plant Germplasm Innovation and Resource Utilization, School of Tea & Food Science and Technology, Anhui Agricultural University, 130 West Changjiang Road, Hefei, Anhui 230036, China; National Key Laboratory for Tea Plant Germplasm Innovation and Resource Utilization, School of Tea & Food Science and Technology, Anhui Agricultural University, 130 West Changjiang Road, Hefei, Anhui 230036, China; National Key Laboratory for Tea Plant Germplasm Innovation and Resource Utilization, School of Tea & Food Science and Technology, Anhui Agricultural University, 130 West Changjiang Road, Hefei, Anhui 230036, China; National Key Laboratory for Tea Plant Germplasm Innovation and Resource Utilization, School of Tea & Food Science and Technology, Anhui Agricultural University, 130 West Changjiang Road, Hefei, Anhui 230036, China; National Key Laboratory for Tea Plant Germplasm Innovation and Resource Utilization, School of Tea & Food Science and Technology, Anhui Agricultural University, 130 West Changjiang Road, Hefei, Anhui 230036, China; National Key Laboratory for Tea Plant Germplasm Innovation and Resource Utilization, School of Tea & Food Science and Technology, Anhui Agricultural University, 130 West Changjiang Road, Hefei, Anhui 230036, China

## Abstract

Fluoride (F) is a nonessential but potentially harmful element for plants, especially when present in excess. The tea plant is known for its ability to hyperaccumulate F from the soil and eventually accumulates in the leaves; however, how the tea plant transports F to the leaves remains unclear. Here, we found that Se can significantly decrease the transport efficiency of F from root to leaf. Therefore, RNA-Sequencing was performed on tea roots cotreated with selenite and fluoride, and then we isolated a plasma membrane-localized F transporter CsNPF2.3 from tea plant roots and examined its role in transport of F in tea plants. The results showed that CsNPF2.3 exhibited F transport activity when heterologously expressed in yeast. Expression pattern analysis revealed that *CsNPF2.3* is expressed in epidermal cells, cortex cells, and xylem parenchyma cells in roots. Overexpression of *CsNPF2.3* in tea roots significantly increased F content in the root, stem, and leaf, and enhanced the transport efficiency of F from root to leaf. Furthermore, in nine tea cultivars, *CsNPF2.3* expression in the root was significantly positively correlated with F content in the leaf and root, and the transport efficiency of F from root to leaf. Altogether, these findings suggest that CsNPF2.3 was involved in uptake and transport of F in tea plants.

## Introduction

F is the smallest and most electronegative halogen, widely distributed in nature [1, 2]. It ranks 13th in abundance in the Earth’s crust and has a hazard index of 12 in the biosphere, making it a widespread and persistent environmental contaminant that is difficult to degrade [1, 2]. F has a dual impact on both animal and human health: while moderate intake can strengthen bones and prevent dental caries, excessive intake can lead to conditions such as dental and skeletal fluorosis [3, 4]. Although no studies shown that F is an essential mineral element for plant growth, it is recognized as a phytotoxin that can negatively affect plant development. In F-sensitive plants, even low concentrations can induce physiological changes such as leaf chlorosis, tip necrosis, and premature leaf abscission [5, 6]. However, certain plants, such as tea plants, exhibit high tolerance to F and can accumulate significant amounts from the environment. Under the same growing conditions, the F content in tea plants can be 10–100 times higher than in other plant species [7, 8], with the majority of F accumulating in mature leaves [9]. The tea plant originated in southwestern China [10]. Tea, produced from fresh tea leaves, is one of the three most widely consumed nonalcoholic beverages globally. However, long-term consumption of tea with elevated F levels poses potential health risks, such as dental and skeletal fluorosis in humans [11, 12]. Thus, understanding the underlying molecular mechanisms governing F absorption and transport in tea plants is of theoretical importance for reducing F levels in tea leaves and mitigating health risks for humans.

The tea plant primarily absorbs F from the soil through its roots, subsequently transporting it to the aerial parts, where it accumulates in the leaves [9, 13]. F is an anion that may be absorbed and transported by plants through anion channels. Previous studies have suggested that anion channels served as major pathways for F uptake in tea roots [14, 15]. Anion channels selectively transport anions across membranes, including nitrate transporters, chloride channels (CLCs), aluminum-activated malate transporters (ALMTs), and slow anion channel-associated homologs (SLAC/SLAHs). It is likely that F is transported through these protein channels. Xing *et al.* [16] identified eight genes in the CLC family, with the expression levels of *CLC1–3* being strongly induced by F exposure, suggesting that the proteins encoded by these genes may participate in F transmembrane transport in tea plants. In addition, Huang *et al.* [17] identified a nitrate transporter, CsNRT/PTR3.1, through transcriptome analysis, proposing that CsNRT/PTR3.1 might play a crucial role in F transmembrane transport. The literature further suggests that these anion channels and transporters involved in anion transport may play a vital role in the transport of F.

Nitrate transporters consist of two main families: NRT1 and NRT2. The NRT1 family, also known as the Nitrate Transporter 1/Peptide Transporter Family (NPFs), is a major subgroup within the PTR family. NPFs are widely distributed in both eukaryotes and prokaryotes and have been identified in various plant species. The substrates transported by NPFs are diverse, including nitrate (NO_3_^−^) [18, 19], amino acids [20], peptides [21, 22], plant hormones [23–25], and glucosinolates [26, 27]. Recent studies have also shown that some NPFs can mediate chloride (Cl^−^) transport. For instance, ZmNPF6.4 and MtNPF6.5, homologs of *Arabidopsis* AtNPF6.3, were found to facilitate Cl^−^ transmembrane transport in *Zea mays* and *Medicago*, respectively, and exhibited high selectivity for Cl^−^ transport [28, 29]. Li *et al.* [30] reported that AtNPF2.4, located in the plasma membrane in the stele cells, and mediated long-distance transport of Cl^−^ in *Arabidopsis*. As an important transporter family, NPFs exhibit broad substrate specificity, with a particular ability to transport Cl^−^. Given that F and Cl are both halogens from the same group and share similarities in their absorption and transport mechanisms, we hypothesize that NPFs may also play a critical role in F transport.

**Figure 1:**
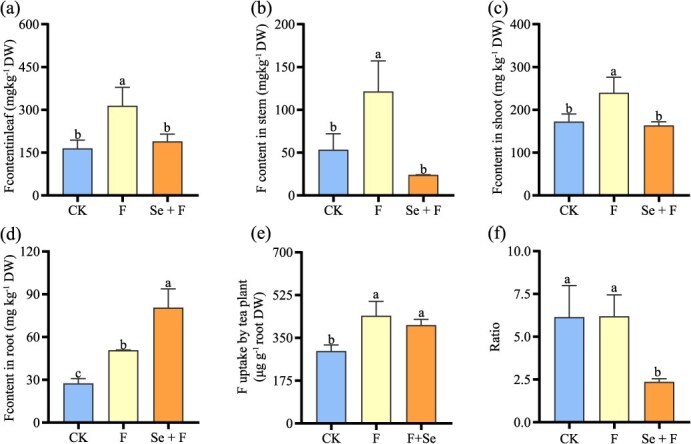
F accumulation, uptake, and translocation in tea plants after F and Se + F treatment. (a), (b), (c), and (d) represent F content in leaf, stem, shoot (leaf + stem), and root, respectively. (e) F uptake by root in tea plant. (f) The ratio of F concentration in the leaf and root. The ratio served as an indicator of the transport efficiency of F from root to leaf. CK is the control group (no F). F is the fluoride treatment group. Se + F is the cotreatment of selenium and fluorine. The data presented are represented as means ± standard deviation (SD) with a sample size of *n* = 3. Different letters are used to indicate significant differences at the *P* < 0.05 level.

In previous studies, we found that Se can significantly reduce the F content in tea leaves, while increasing F content in the roots [31]. We speculated that Se regulated expression of some genes encoding F transporters, which could reduce F transport from root to leaf in tea plants. However, there is no literature reporting transporters mediating F transport in the roots. Thus, we conducted transcriptome analysis on tea seedlings cotreated with Se and F to identify differentially expressed genes (DEGs). The transporters encoded by these DEGs may be involved in F transport. From the transcriptome data, we identified a key DEG, *CsNPF2.3*, which is specifically expressed in tea roots. The transporter encoded by *CsNPF2.3* shares a close evolutionary relationship with VviNPF2.1 and VviNPF2.2. Notably, VviNPF2.2 located on the plasma membrane functions in Cl^−^ efflux and modulates shoot anion concentration in *Arabidopsis* [32]. Thus, we speculated that CsNPF2.3 may be involved in transmembrane transport of F in tea plants. To investigate this, we employed a yeast heterologous expression system and tea plant hair root transgenic technology to explore the function of CsNPF2.3 in F transport in tea plants.

**Figure 2:**
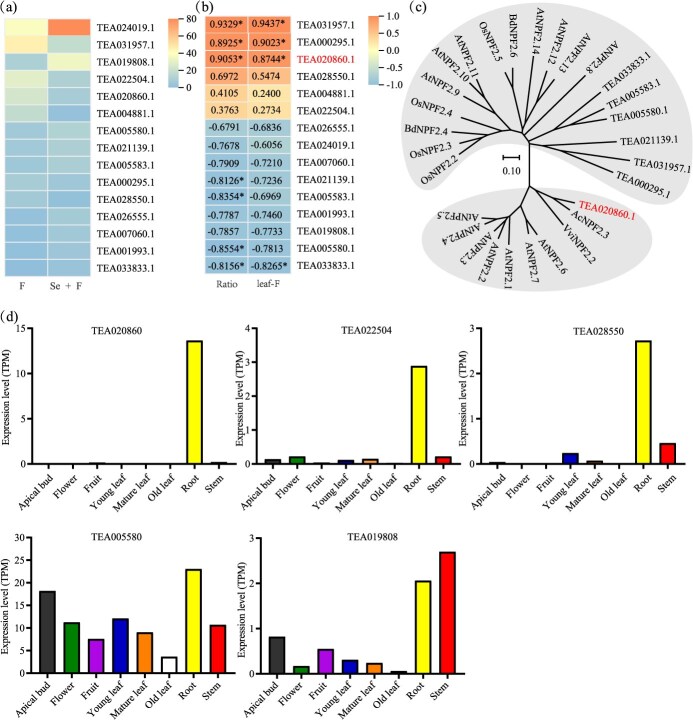
Analysis of DEGs in tea plant roots under F and Se + F treatment. (a) The expression levels of DEGs under F and Se + F treatment in tea plant roots. (b) Pearson correlation analysis between DEG expression and F transport efficiency (ratio), and leaf F content. The ratio served as an indicator of the efficiency of F transport from root to leaf. The leaf-F was F content in leaves. The numbers in the figure represented the correlation coefficients between DEG expression levels and F transport efficiency (root-to-leaf), and leaf F content. ‘*’ indicates significant difference at *P* < 0.05. (c) Phylogenetic tree of CsNPF2.3 with NPF2s in *A. thaliana* (At), *O. sativa* (Os), *V. vinifera* (Vvi), *Actinidia chinensis* (Ac), and *B. distachyon* (Bd). The phylogenetic tree was constructed using MEGA11 with Neighbor-Joining method. The scale showed substitution distance. (d) Expression levels of DEGs encoding nitrate transporter family in eight tissues in tea plants. The data are sourced from the Tea Tree Genome website (Tea Plant Information Archive (TPIA): A comprehensive knowledge database for tea plant (teaplants.cn)).

## Results

### Effect of exogenous Se on F accumulation, uptake, and translocation from root to shoot in tea plants

A previous study found that exogenous Se significantly reduced F content in tea leaves [31]. To investigate the mechanism of Se reducing F content in tea leaves, we measured the F content in roots, stems, and leaves, as well as the F uptake and transport efficiency. The effects of Se on F content in root, stem, and leaf were shown in Fig. 1a, b, and d. The addition of F significantly increased F levels in the roots, stems, and leaves compared to the CK (control, no F). Compared to the treatment with F alone, the Se + F treatment resulted in a significant (*P* < 0.05) reduction in F levels by 39.6% and 80.3% in the leaves and stems, respectively, while increasing by 58.9% in the roots. The results of the analysis on F uptake, translocation, and accumulation in the tea plant under the various treatments are presented in Fig. 1c, e, and f. Compared with the CK, the addition of F significantly elevated (*P* < 0.05) shoot F content. Compared to the F treatment, the addition of Se resulted in a significant 30% decrease in shoot F content (Fig. 1c). The uptake of F by tea roots significantly increased with both F and Se + F treatment compared to the CK. However, the uptake of F did not show a remarkable difference between Se + F treatment and F alone treatment (Fig. 1e), indicating that the addition of exogenous Se did not affect the uptake of F by root in tea plants. Transport efficiency, which represents the ratio of F concentration in the leaf to the root, indicates the plant’s capacity to translocate F from the root to the leaf. Compared with the CK treatment, F treatment did not markedly affect the transport efficiency of F from root to leaf. However, compared with F alone treatment, Se + F treatment markedly reduced transport efficiency of F from root to leaf (Fig. 1f). These results suggest that the reduction in F content in tea leaves caused by Se might be due to Se hindering the translocation of F from root to leaf.

**Figure 3:**
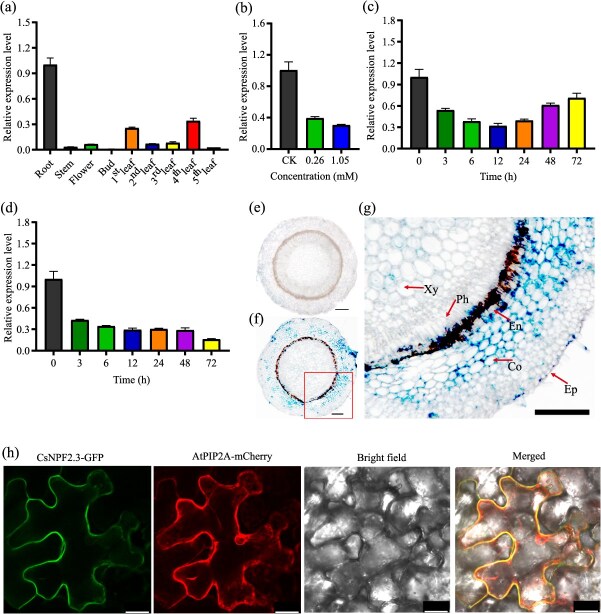
Expression pattern and subcellular localization of *CsNPF2.3*. (a) Relative expression level of *CsNPF2.3* in different tissues of tea plants. (b) Relative expression level of *CsNPF2.3* under different F concentration treatment for 24 h. (c–d) Relative expression level of *CsNPF2.3* with different time under 0.26 and 1.05 mM F treatment, respectively. (e–g) *In situ* PCR analysis of *CsNPF2.3* in tea root. (e) Negative control. (f) Expression location of *CsNPF2.3*. (g) The magnified areas of the red box in (f). The stained area represents cells of gene expression. Ep, epidermis; Co, cortex; En, endodermis; Ph, phloem; Xy, xylem. (h) Subcellular localization of CsNPF2.3 in epidermal cells of tobacco. Fluorescence signals from GFP, mCherry, and the merged and bright-field images are shown. All data are means ± SD, at least three biological replicates (*n* ≥ 3). Bars in (e) are 0.5 μm, 1 mm in (f, g), and 25 μm in (h).

### Identification and screening of target genes

Transcriptome sequencing analysis indicated that 2535 genes were upregulated, while 2919 genes were downregulated in tea roots (Fig. S1). In the F versus (Se + F), 15 DEGs were annotated as genes encoding NPF transporters, with nine genes being upregulated and six genes being downregulated (Fig. 2a and Table S2). Pearson correlation analysis between the DEGs and leaf F content indicated that the expression levels of four genes were significantly correlated with F content in the leaves, including *TEA031957.1*, *TEA000295.1*, *TEA020860.1*, and *TEA033833.1* (Fig. 2b and Table S3). In addition, an analysis of the relationship between DEG expression levels and the ratio of F content in leaf to root revealed that seven genes were significantly correlated with the ratio (Fig. 2b and Table S3). Of these, three genes exhibited a significant positive correlation, while four genes showed a significant negative correlation. These DEGs may play crucial roles in the translocation of F from root to leaf. According to the data from the tea plant genomic database (Fig. 2d and Fig. S2), five genes (*TEA028550.1*, *TEA019808.1*, *TEA005580.1*, *TEA020860.1*, and *TEA022504.1*) were found to be highly expressed in the roots. Phylogenetic analysis showed that the protein encoded by *TEA020860.1* shares 76% identity with AcNPF2.3, suggesting a close evolutionary relationship (Fig. 2c). Thus, TEA020860.1 was named as CsNPF2.3.

**Figure 4:**
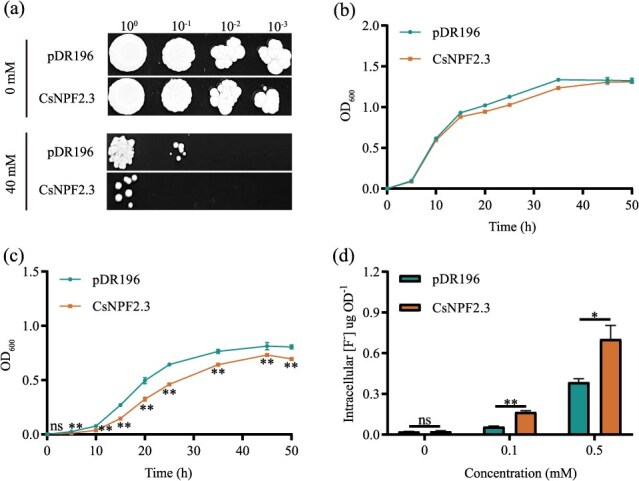
F transport activity of CsNPF2.3. (a) The yeast strain BY4743 transformed with pDR196 or CsNPF2.3 was serially diluted and grown on the YPD media containing 0 or 40 mM F for 5 days. (b) and (c) were the growth rates of yeast strains in liquid YPD medium containing 0 and 40 mM F, respectively. ‘ns’ indicated no significant difference. ‘*’ and ‘**’ indicated significant differences at the *P* < 0.05 and *P* < 0.01 level between pDR196 and CsNPF2.3 at the same time. (d) F content in yeast cells transformed with pDR196 or CsNPF2.3. Yeast cells were incubated in liquid YPD containing 0, 0.1, or 0.5 mM F for 24 h, and the F content in yeast cells was measured. ‘ns’ indicated no significant difference. ‘*’ and ‘**’ indicated significant differences at the *P* < 0.05 and *P* < 0.01 level between pDR196 and CsNPF2.3 at the same F concentration, respectively. All data are means ± SD, at least three biological replicates (*n* ≥ 3).

### Expression pattern and tissue localization of CsNPF2.3 in tea plants

We examined the expression pattern of *CsNPF2.3* using quantitative real-time–polymerase chain reaction (qRT-PCR). Under normal field growth conditions, *CsNPF2.3* expression was analyzed across various tissues, including roots, stems, buds, flowers, and leaves (from the first to fifth leaves). The results indicated that *CsNPF2.3* was predominantly expressed in the roots, with minimal expression detected in other tissues (Fig. 3a), which is consistent with the results published in genomic databases (Fig. 2d). When 1-year-old tea cuttings were grown in a nutrient solution containing 0, 0.26, or 1.05 mM F for 3, 6, 12, 24, 48, and 72 h, F treatment significantly downregulated *CsNPF2.3* expression in the roots. The reduction in *CsNPF2.3* expression was concentration-dependent, with the 1.05 mM F treatment causing a more pronounced decrease compared to the 0.26 mM F treatment (Fig. 3b). Under the 0.26 mM F treatment, *CsNPF2.3* expression initially decreased before increasing over time (Fig. 3c). However, under the 1.05 mM F treatment, a continuous and significant decrease in *CsNPF2.3* expression was observed (Fig. 3d). To further analyze the cell-specific expression of *CsNPF2.3* in tea plant roots, *in situ* PCR was performed to localize its expression (Fig. 3e–g). Compared to the control, *CsNPF2.3* signals were detected in the epidermal cells, cortex cells, and xylem parenchyma cells of the roots, with the strongest expression observed in the cortex cells (Fig. 3g). These results suggest that *CsNPF2.3* is expressed in epidermal cell, cortex cell, and xylem parenchyma cells of root, with the highest expression in cortex cells.

### *CsNPF2.3* encodes a transporter located on the plasma membrane

To determine the subcellular localization of CsNPF2.3, we constructed a vector expressing a CsNPF2.3-GFP (Green Fluorescent Protein) fusion protein driven by the 35S promoter, which was subsequently introduced into tobacco epidermal cells. Fluorescence from GFP was observed at the plasma membrane, as evidenced by its colocalization with the plasma membrane marker AtPIP2A-mCherry, which was fused to red fluorescent protein (Fig. 3h). These observations confirm that *CsNPF2.3* encodes a protein localized to the plasma membrane.

### F transport activities of CsNPF2.3 in yeast

To assess whether CsNPF2.3 exhibits F transport activity, we heterologously expressed it in yeast strains. On solid yeast extract peptone dextrose (YPD) medium without F (0 mM F), the growth of yeast strains expressing *CsNPF2.3* showed no difference compared to strains transformed with the empty vector *pDR196*. However, in the presence of 40 mM F, yeast strains expressing *CsNPF2.3* exhibited more pronounced growth suppression compared to those expressing *pDR196* (Fig. 4a). Similarly, growth curves for yeast strains expressing *CsNPF2.3* and *pDR196* in liquid medium showed no difference in the absence of F (Fig. 4b). Under 40-mM F treatment, the growth of strains expressing *CsNPF2.3* was significantly reduced compared to strains expressing *pDR196* (Fig. 4c). Furthermore, strains expressing *CsNPF2.3* accumulated ~3-fold and 2-fold more F than those expressing *pDR196* when exposed to 0.1 and 0.5 mM F, respectively (Fig. 4d). These results indicate that CsNPF2.3 possesses F transport activity in yeast. In contrast, no transport activity for NO_3_^−^, Cl^−^, or Br^−^ was detected when *CsNPF2.3* was expressed in yeast under our experimental conditions (Fig. S3).

### Functional analysis of CsNPF2.3 in hairy roots of tea plants

**Figure 5:**
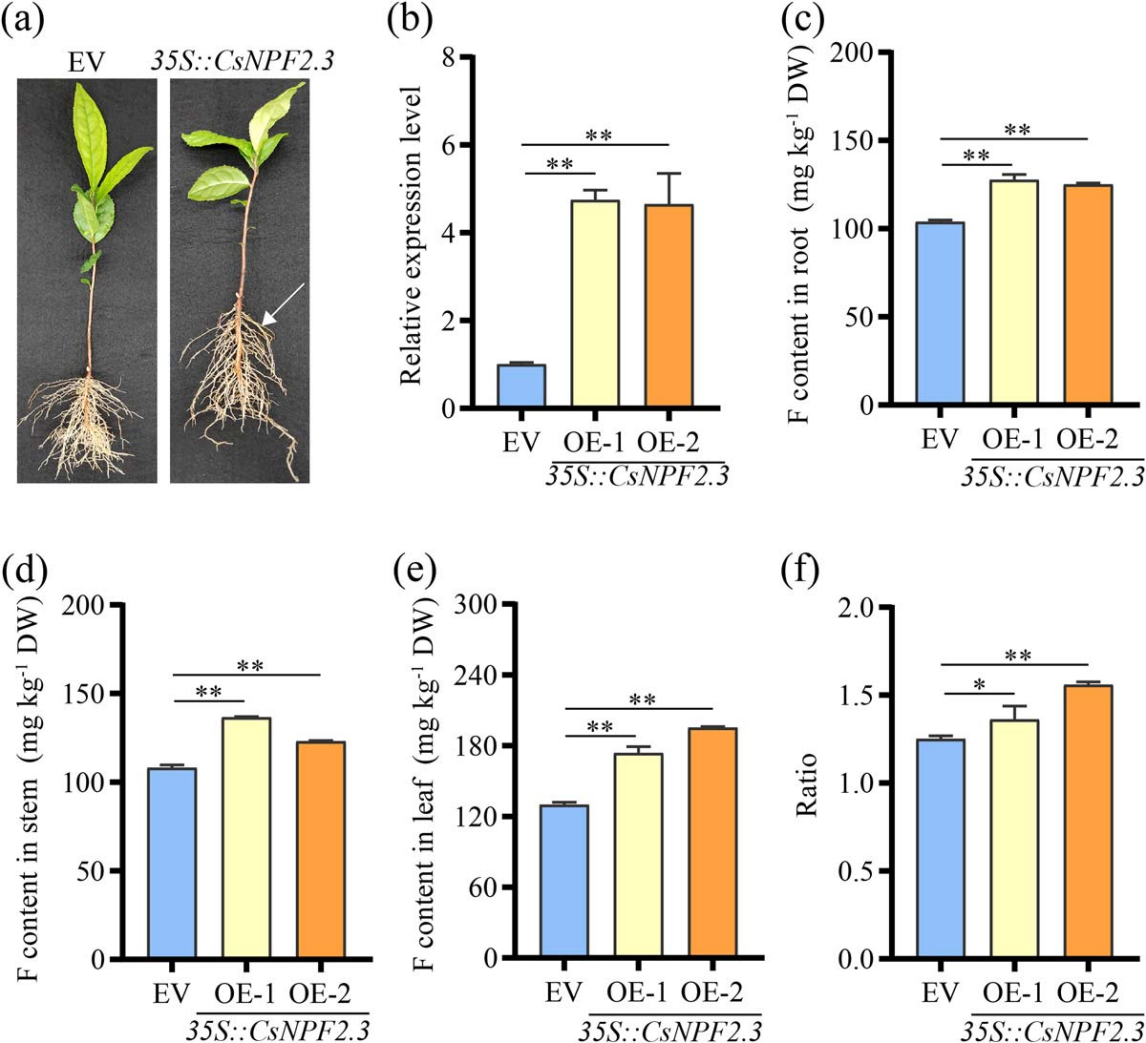
Functional characterization of CsNPF2.3 in hairy roots of tea plants. (a) Representative images of transgenic tea hairy roots expressing *CsNPF2.3* and empty vector. White arrows indicated the hairy roots. (b) Relative expression level of *CsNPF2.3* in EV and CsNPF2.3 hairy roots. The relative expression levels were computed through the 2^-ΔΔCT^ method. (c) F content of primary roots in EV and CsNPF2.3 tea plant. (d) F content of stems in EV and CsNPF2.3 tea plant. (e) F content of leaf in EV and CsNPF2.3 tea plant. (f) The ratio of F content in leaves and roots in EV and CsNPF2.3 tea plant. ‘EV’ represented the tea hairy roots line of transgenic empty vector. ‘*35S::CsNPF2.3*’ represented the activation of *CsNPF2.3* gene expression with CaMV *35S* as the promoter. OE-1 and OE-2 represented the overexpression of *CsNPF2.3* in transgenic hairy root system, respectively. ‘*’ and ‘**’ indicated significant differences at the *P* < 0.05 and *P* < 0.01 level, respectively. All data are means ± SD, at least three biological replicates (*n* ≥ 3).

Currently, the lack of a stable genetic transformation system for tea plants has made it difficult to generate stable genetic lines. However, Li *et al.* [33] reported that the hairy root transformation system can be used to study gene function in tea plants. To investigate the role of CsNPF2.3 in F transport, we employed this system to create *CsNPF2.3*-overexpressing tea plants. qRT-PCR analysis showed that *CsNPF2.3* transcript levels were significantly higher in transgenic plants compared to those with the empty vector (Fig. 5b). The F content in the root, stem, and leaf of *CsNPF2.3*-overexpressing tea plants was markedly increased compared to the empty vector controls (Fig. 5c–e). Since F is primarily transported from the roots to the leaves, we used the ratio of F content in the leaves to that in the roots as an indicator of root-to-leaf transport efficiency. The results indicated that the ratio was dramatically higher in *CsNPF2.3*-overexpressing plants compared to controls (Fig. 5f), suggesting that CsNPF2.3 plays a crucial role in F transmembrane transport.

### Expression level of *CsNPF2.3* in roots and F content in roots and leaves of different tea plant cultivars

**Figure 6:**
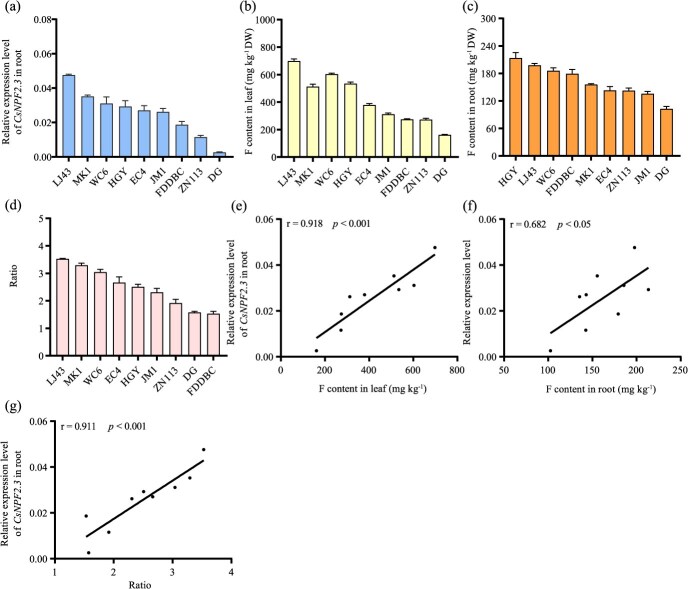
Analysis of *CsNPF2.3* expression and F content in different tea plant cultivars. (a) Relative expression level of *CsNPF2.3* in tea roots. Relative expression was represented with 2^-ΔCT^. (b) F content in tea leaves. (c) F content in tea roots. (d), Ratio of F content in leaf and root. (e) Pearson correlation analysis between the relative expression of *CsNPF2.3* and F content in tea leaves. (f) Pearson correlation analysis between the relative expression of *CsNPF2.3* and F content in tea roots. (g) Pearson correlation analysis between the relative expression of *CsNPF2.3* and ratio of F content in leaf and root. Longjing 43, LJ43; Mingke 1, MK1; Wancha 6, WC6; Huangguanyin, HGY; Echa 4, EC4; Jiaming 1, JM1; Fuding Dabaicha, FDDBC; Zhenong 113, ZN113; Dangui, DG. All data are means ± SD, at least three biological replicates (*n* ≥ 3).

To further elucidate the role of CsNPF2.3 in F transport in tea plants, we assessed the expression level of *CsNPF2.3* in roots and measured F content in the leaves and roots across nine different tea cultivars (Fig. 6). We observed significant variations in *CsNPF2.3* expression levels in the roots among the different cultivars (Fig. 6a). Correspondingly, F content in leaves and roots, and ratio of leaf F content to root F content varied significantly across these cultivars (Fig. 6b–d). Pearson correlation analysis revealed that the expression level of *CsNPF2.3* was significantly positively correlated with root F content (*r* = 0.682, *P* < 0.05) and leaf F content (*r* = 0.918, *P* < 0.001) (Fig. 6e and f), respectively. In addition, the expression level of *CsNPF2.3* was significantly positively correlated with the ratio of F content in leaf to root (*r* = 0.911, *P* < 0.001) (Fig. 6g). These results further indicate that CsNPF2.3 plays a crucial role in F transport in tea plants.

## Discussion

F is primarily absorbed by the roots of tea plants, transported to the aerial parts, and eventually accumulates in the leaves [9]. The transmembrane transport of F in roots determines F accumulation in tea leaves. Current research has identified several F transporters involved in the transmembrane transport of F in tea leaves [34–36]. However, no studies have yet reported F transporters involved in the uptake and transport of F in the roots. Previous study indicated that exogenous addition of Se in nutrient solution can significantly reduce the F content in tea leaves [31]. By further measuring the transport capacity of F from root to leaf, we found that Se significantly reduced F transport from root to leaf (Fig. 1f), suggesting that Se may regulate F-related transporters in the root to mediate F transport from root to leaf. To investigate the molecular mechanisms of F absorption and transport in roots, we utilized the cotreatment of Se and F to identify F-related transporters highly expressed in the roots through transcriptome sequencing in this study. The Nitrate Transporter 1/Peptide Transporter Family (NPF) is a relatively large group of anion transporters involved in the transport of a diverse substrates, such as NO_3_^−^, IAA, ABA, jasmonic acid, and gibberellins [23, 25, 29]. Recent studies also reported the involvement of NPFs in the uptake and transport of Cl^−^ [28–30, 32]. Given that F and Cl are in the same group in the periodic table, they may share similarities in their uptake and transport processes in plants. We identified a key candidate gene, *CsNPF2.3*, from the transcriptome (Fig. 2a, b, d and Fig. S2). And CsNPF2.3 showed 57% identity with VviNPF2.2 (Fig. 2c), which is involved in Cl^−^ efflux in the root of *Vitis vinifera* L. [32]. This provides us with additional evidence that CsNPF2.3 may be involved in F transport in tea plants. The results of heterologous expression in yeast indicated that CsNPF2.3 exhibited F transport activity (Fig. 4). Therefore, CsNPF2.3 may be involved in F transport in tea plants.

*CsNPF2.3* is specifically expressed in the roots (Fig. 3a), suggesting its potential involvement in F transport within the root system. Mineral elements absorbed by plant roots are typically transported radially through both symplastic and apoplast pathways [37]. During these two processes, the import and export of elements into cells and organelles rely on membrane-localized transporters to mediate these processes. Like other mineral elements, F also needs to be transported through both the symplastic and apoplastic pathways. *CsNPF2.3* encodes a plasma membrane-localized transporter, which is expressed in the epidermal cells, cortex cells, and xylem parenchyma cells of tea roots (Fig. 3e–h). And *CsNPF2.3* overexpression in tea root significantly increased F content in the root, stem and leaf (Fig. 5c–e), and the ratio of F content in the leaf and root (Fig. 5f). Thus, CsNPF2.3 may be involved in the retrieval of F from the apoplast and the uptake of F from the soil solution. In addition, *CsNPF2.3* expression was highly positively correlated with F content in root and leaf, and F transport from root to leaf (Fig. 6e–g), further suggesting that CsNPF2.3 may be involved in retrieval of F from the apoplast and the uptake of F from the soil solution in tea plants. However, the expression level of *CsNPF2.3* was inhibited by F (Fig. 3b), which may be a stress response of tea roots to exogenous F. Since F is not an essential nutrient for plant growth and is regarded as a common phytotoxin [38], tea roots exposed to F may downregulate *CsNPF2.3* expression to alleviate F stress in the short term.

In conclusion, our results demonstrated that the plasma membrane-localized CsNPF2.3 may exhibit F transport activity. Overexpression of *CsNPF2.3* significantly increased F content in roots, stems, and leaves. Moreover, the expression of *CsNPF2.3* in the root was strongly positively correlated with F transport from root to leaf. Based on these findings, we hypothesize that CsNPF2.3 is involved in retrieval of F from the apoplast and uptake of F from the soil solution. Given the detrimental effects of excessive F on both tea plants and human health, the identification of CsNPF2.3 provides a theoretical foundation for future efforts to screen for low-F cultivars of tea plants.

## Materials and methods

### Plant materials and treatment

One-year-old cuttings, obtained from Dechang Seedling Company in Shucheng Country, Anhui Province, China, were hydroponically cultured in a greenhouse under natural sunlight at a temperature of 25°C during the day and 20°C at night. The seedlings were grown in a basic nutrient solution as described by Konishi [39]. After the development of lateral roots, tea plants of uniform size were selected for further studies.

Tea plants of uniform size were transferred to containers (5 plants pot^−1^) containing 2 l of nutrient solution with 0 (nutrient solution, control), 0.26 mM F (NaF, Sinopharm Chemical Reagent Co., Ltd, China), and 0.26 mM F + 0.006 mM Se (Na_2_SeO_3_, Nanjing Chemical Reagent Co., Ltd, China) for 3 weeks. Each treatment has three replicates. Three weeks after F and Se treatment, leaves, stems, and roots were harvested from each treatment group and stored at −80°C. Some samples are used to determine the effect of Se on F uptake and translocation, while others are used for transcriptome analysis. In addition, the roots of tea plants were treated with 0, 0.26, and 1.05 mM F for 0, 3, 6, 12, 24, 48, and 72 h and were harvested for expression analysis.

Tea seeds were sown in plastic pots containing a mixture of soil (25%), pearlite (25%), and vermiculite (50%). The pots were maintained in a greenhouse under natural sunlight with the same day/night temperature regime (25°C/20°C) and irrigated weekly with tap water. After ~4 months of growth, healthy plants of similar size were selected for transgenic hairy root assays.

On 1 November 2023, leaves (1–3 leaves) and lateral roots were collected from nine tea plant cultivars grown in the Germplasm Resource Garden (Guohe Town) of the State Key Laboratory of Tea Plant Biology and Resource Utilization, Anhui Agricultural University. The cultivars included ‘Longjing 43 (LJ43)’, ‘Mingke 1 (MK1)’, ‘Wancha 6 (WC6)’, ‘Huangguanyin (HGY)’, ‘Echa 4 (EC4)’, ‘Jiaming 1 (JM1)’, ‘Fuding Dabaicha (FDDBC)’, ‘Zhenong 113 (ZN113,)’, and ‘Dangui (DG)’. All samples were immediately frozen in liquid nitrogen and stored at −80°C for further analysis.

### Transcriptome analysis of tea plants under F and Se treatment

The extraction of total RNA from root samples of ‘Shuchazao’ was conducted using the Total RNA Extraction (Trizol) kit from Invitrogen, USA, according to the manufacturer’s instructions. After confirming the quality of the RNA, cDNA libraries were prepared and subjected to sequencing using the Illumina sequencing platform, performed by Metware Biotechnology Co., Ltd. (Wuhan, China). The reads of all samples were mapped to the reference genomes of *Camellia sinensis* cv. Shuchazao [40] using HISAT (version 2.1.0) software. The expression levels of genes were calculated utilizing the featureCounts software (version: 1.6.2) with the Fragments Per Kilobase Per Million reads (FPKM). Significant DEGs were chosen based on the following criteria: a false discovery rate (FDR) value below the threshold (Q < 0.05) and |log_2_ (Fold change)| ≥ 0.58.

### Phylogenetic analysis of CsNPF2.3

Multiple amino acid sequence alignments were conducted using MEGA 11. The proteins included in the alignment were from *Arabidopsis thaliana*, *Oryza sativa*, *V. vinifera*, and *Brachypodium distachyon*. Protein sequences from various plant species were retrieved from the NCBI database (https://www.ncbi.nlm.nih.gov/). A neighbor-joining phylogenetic tree was constructed with 1000 bootstrap replicates to assess branch support.

### Subcellular localization of CsNPF2.3

The open reading frame (ORF) of *CsNPF2.3* without the stop codon was amplified using gene-specific primers (Table S1) and cloned into the pCAMBIA1305.1-*GFP* vector under control of the *CaMV 35S* promoter using primers *CsNPF2.3-GFP-F* with SpeI site and *CsNPF2.3-GFP-R* with BamHI site. The recombinant plasmid was introduced into *Agrobacterium rhizogenes* GV3101. The plasma membrane marker *AtPIP2A-mCherry* [41] and *CsNPF2.3-GFP* were transiently expressed in *Nicotiana benthamiana* leaves. After transformation, the plant was placed in the dark at room temperature for a day. GFP fluorescence was visualized using laser confocal microscopy (LEICA DMi8, Germany).

### *In situ* PCR analysis

*In situ* PCR was conducted following the method described by Lin *et al.* [42] Briefly, young primary roots were sectioned into appropriate sizes, fixed in FAA solution (63% ethanol, 5% acetic acid, 2% formaldehyde), vacuum infiltrated for 20 min, and then stored at 4°C for 12 h. The tissues were washed three times with 63% ethanol and 5% acetic acid for 10 min each, followed by two washes with 1× PBS for 5 min each. Samples were embedded in 5% low-melting point agarose and sectioned using a Leica RM2255 microtome (Leica, Nussloch, Germany). The sections were washed twice with RNase-free water and treated with 3 μg/ml proteinase K at 25°C for 30 min. Proteinase K was inactivated at 85°C for 2 min, followed by washes with 1× PBS and RNase-free water for 5 min each. Genomic DNA was removed using 1 U/μl DNase I at 37°C overnight. DNase I activity was halted with 15 mM EDTA (pH 8.0) at 75°C for 10 min, followed by two washes with RNase-free water. Reverse transcription was performed using the PrimeScript II 1st Strand cDNA Synthesis Kit (TAKARA, Cat. No. 9750). PCR was carried out using cDNA as the template, with the reaction conditions listed in Supplementary Table S1. After PCR, the tissue sections were washed twice with 1× PBS for 5 min each and blocked with a confining solution containing 0.1% BSA and 5% skim milk for 30 min. The sections were then incubated with an alkaline phosphatase-conjugated antibody (dilution 1:500) for 1 h, followed by two washes with 10× washing buffer for 15 min each. For visualization, the tissue was stained with BM Purple AP substrate for 1 h, washed twice with RNase-free water, and then imaged under a microscope.

### *CsNPF 2.3* expression in yeast cells

The ORF of *CsNPF2.3* was amplified using gene-specific primers (Supplement Table S1) and cloned into *pDR196* vector using primers *pDR196-CsNPF2.3-F* with SpeI site and *pDR196- CsNPF2.3-R* with EcoRI site. The resulting *pDR196-CsNPF2.3* plasmid and the empty vector *pDR196* were introduced into the *Saccharomyces cerevisiae* strain BY4743. Transformants were selected on Yeast Nitrogen Base (YNB-ura) plates and confirmed by PCR. For F tolerance analysis of the *CsNPF2.3* transformants, the yeast suspension was diluted to final OD_600_ concentrations of 10^0^, 10^−1^, 10^−2^, and 10^−3^. Aliquots (2.5 μl) of each dilution were spotted on solid YPD medium containing various concentrations of F (0 and 40 mM), Cl (500 and 800 mM), NO_3_^−^ (400, 600, and 800 mM) and Br (500 and 800 mM), respectively. Plates were incubated at 28°C and photographed. Meanwhile, positive strains with an OD_600_ of 0.8 were inoculated into YPD liquid medium with 0, 20, 40, and 60 mM F. Cultures were grown on a shaking incubator at 220 rpm and 28°C. Optical density at 600 nm (OD_600_) was measured every 5 h until 50 h using a Microplate reader (Spectra Max M2) to construct the growth curve. For transport activity assay of F, the yeast carrying *pDR196-CsNPF2.3* or *pDR196* was cultured in liquid medium containing 0.1 and 0.5 mM F. The samples were harvested and washed with deionized water after 24 h. F in yeast was extracted with the method provided by Song *et al.* [35]

### Transformation of hairy roots in tea plant

For tea plant hairy root transformation, the *CsNPF2.3-GFP* construct was introduced into *Agrobacterium rhizogenes* strain ATCC 15834 by electroporation. The confirmed positive transformants were then used to infect ~4-month-old tea seedlings (‘Shuchazao’). Prior to infection, lateral roots were removed, and the primary roots were wounded to facilitate transformation. The seedlings were inoculated with the transformed *A. rhizogenes* ATCC 15834 strains. After 3 months of growth, the transformed seedlings were treated with a nutrient solution containing 0.26 mM F for 24 h. Following treatment, roots, stems, and leaves were rapidly frozen in liquid nitrogen and stored at −80°C for further analysis.

### *CsNPF2.3* expression analyses

For tissue-specific expression analysis, total RNA was extracted from roots, stems, flowers, buds, and leaves (from the first to fifth leaves). For expression of *CsNPF2.3* in different cultivars, different F concentration treatment, and different time treatment in the same F concentration, total RNA was extracted from roots. First-strand cDNA synthesis was performed using the HiScript III All-in-One RT SuperMix Perfect for qPCR (Vazyme, China). qRT-PCR was conducted using gene-specific primers (Table S1). The data were analyzed with Opticon Monitor software (Bio-Rad). The relative expression levels of *CsNPF2.3* were normalized against the internal reference gene *CsGAPDH*. Unless otherwise specified, the relative expression levels were computed through the 2^-ΔΔCT^ method.

### Extraction and measurement of F in Samples

The roots, stems, and leaves of the tea plant were dried at 105°C until a constant weight was achieved, after which they were ground into a fine powder using a grinder. Sample pretreatment for determining total F content followed the method described by Shyu *et al.* [43] F concentration in both plant and yeast samples was measured using a fluoride ion-selective electrode.

### Data analysis

Statistical analysis was performed using one-way ANOVA, with significance levels set at *P* < 0.05 and *P* < 0.01. All statistical analyses were conducted with SPSS 26 software, while graphs and visual representations were generated using GraphPad Prism 8. Data are presented as means ± SD, with a minimum of three biological replicates (*n* ≥ 3). The F contents (C_root-F_, C_leaf-F_, and C_shoot-F_) in roots, leaf, and shoots were computed based on dry weight. Total F (T_F_) and transfer factor (TF) was computed using the following equations. (1)–(5): 


(1) \begin{equation*} \mathrm{T}_{\text{root-F}}=\mathrm{C}_{\text{root-F}}\times\mathrm{Root}_{\mathrm{Dry biomass}} \end{equation*}

(2) \begin{equation*} \mathrm{T}_{\text{shoot-F}}=\mathrm{C}_{\text{shoot-F}}\times\mathrm{shoot}_{\mathrm{Dry biomass}} \end{equation*}

(3) \begin{equation*} \mathrm{T}_{\mathrm{F}}=\mathrm{T}_{\text{root-F}}+\mathrm{T}_{\text{shoot-F}} \end{equation*}

(4) \begin{equation*} \mathrm{F}_{\mathrm{uptake}}=\mathrm{T}_{\mathrm{F}}/\mathrm{Root}_{\mathrm{Dry biomass}} \end{equation*}

(5) $$ \begin{equation*} \mathrm{Ratio}=\mathrm{C}_{\text{leaf-F}}/\mathrm{C}_{\text{roof-F}}. \end{equation*}

## Supplementary Material

Web_Material_uhaf072

## Data Availability

All relevant data in this study are incorporated into the article and its supplementary file.
