# Supplementary material for: Root-specific expression of CsNPF2.3 is involved in modulating fluoride accumulation in tea plant (Camellia sinensis)
Source: Hortic Res. 2025 Mar 3;12(6):uhaf072. doi: 10.1093/hr/uhaf072 (PMC12038894; doi:10.1093/hr/uhaf072)
Supplement: Web_Material_uhaf072 [file web_material_uhaf072.zip › Supplementary Figure.docx]

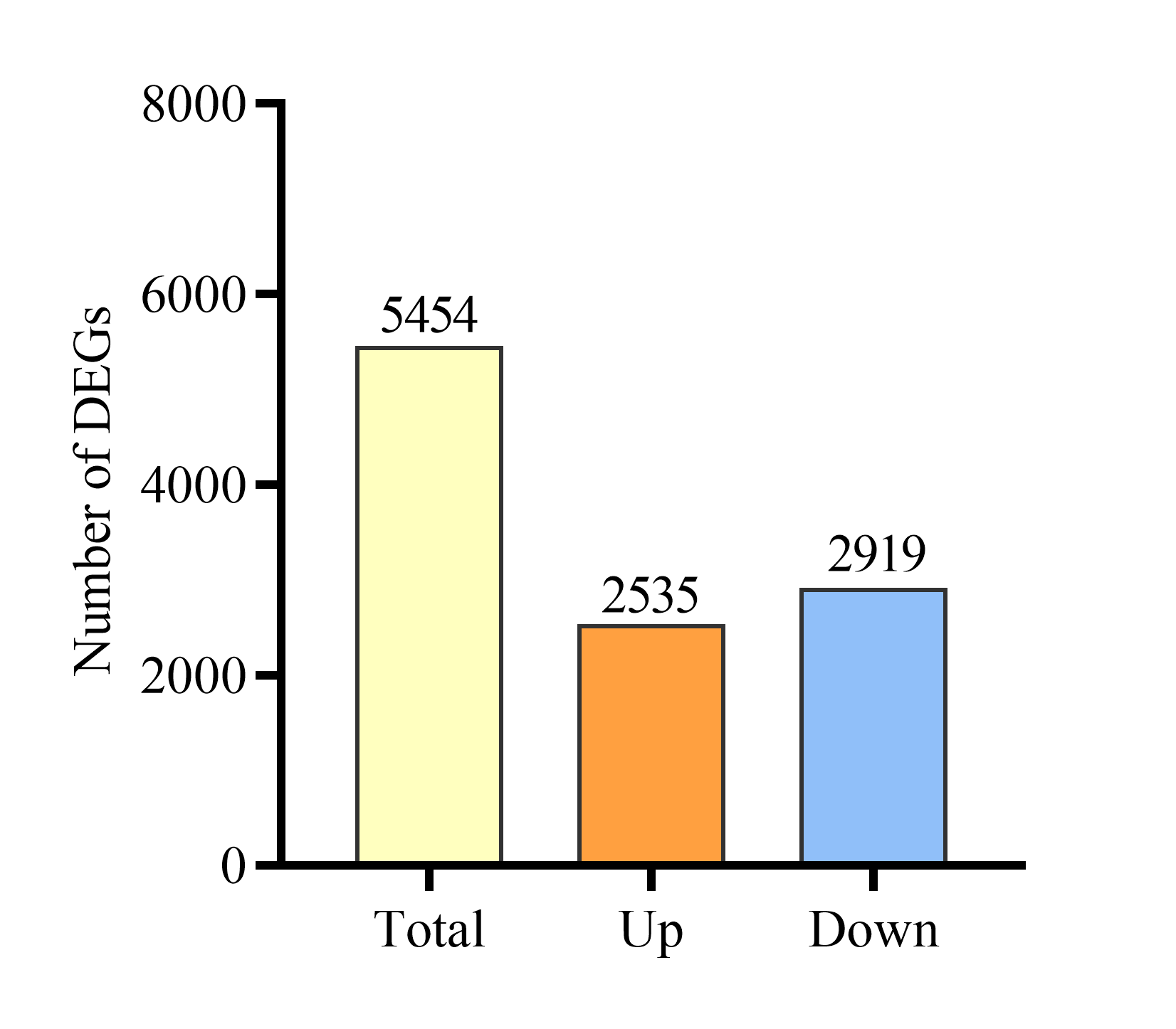


Figure S1. All DEGs in tea plant roots between F and Se + F.


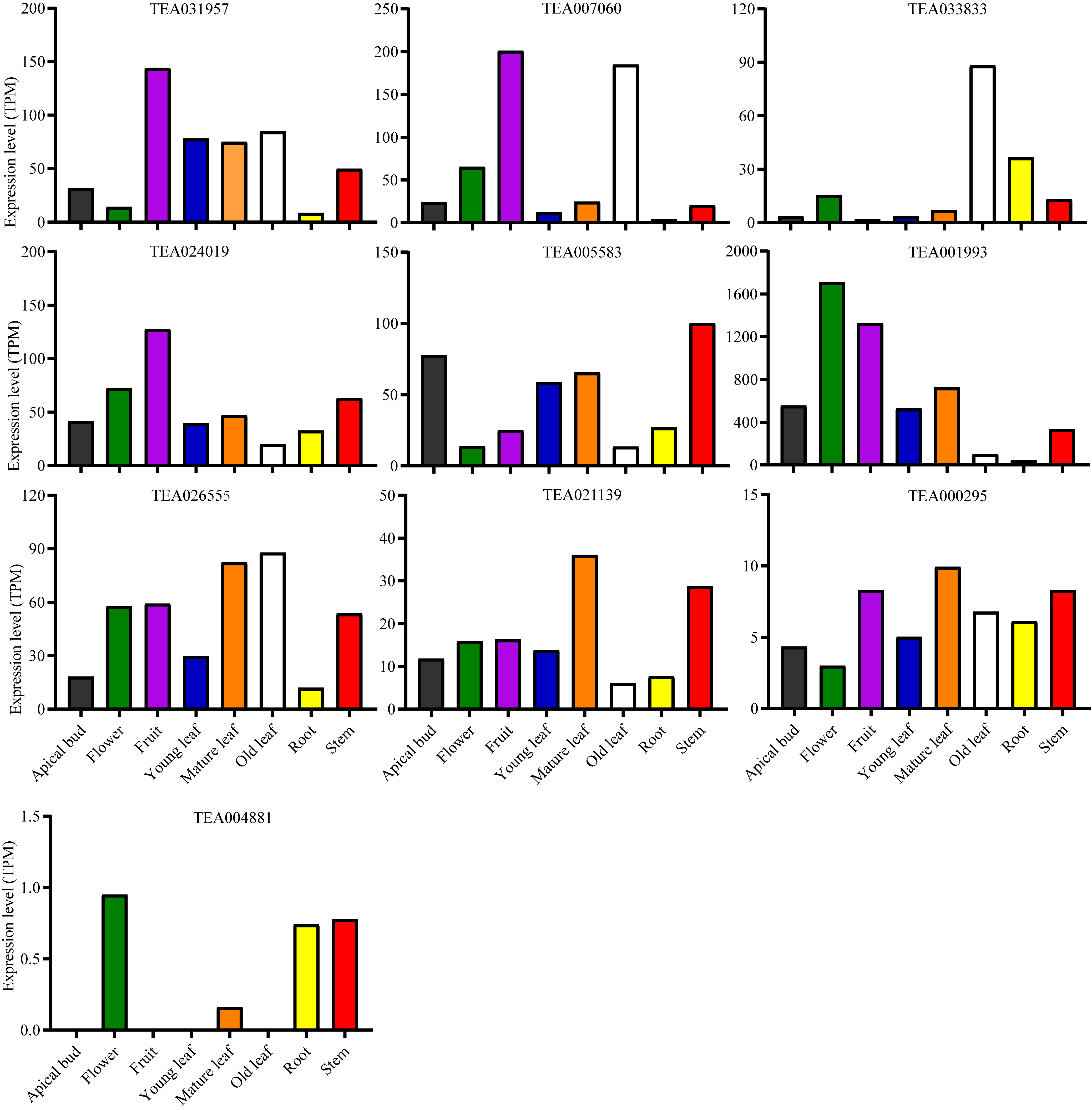


Figure S2. Expression levels of differentially expressed genes encoding nitrate transporter family in 8 tissues in tea plant. The data is sourced from the Tea Tree Genome website ([Tea Plant Information Archive(TPIA): A comprehensive knowledge database for tea plant. (teaplants.cn)](http://tpia.teaplants.cn/)


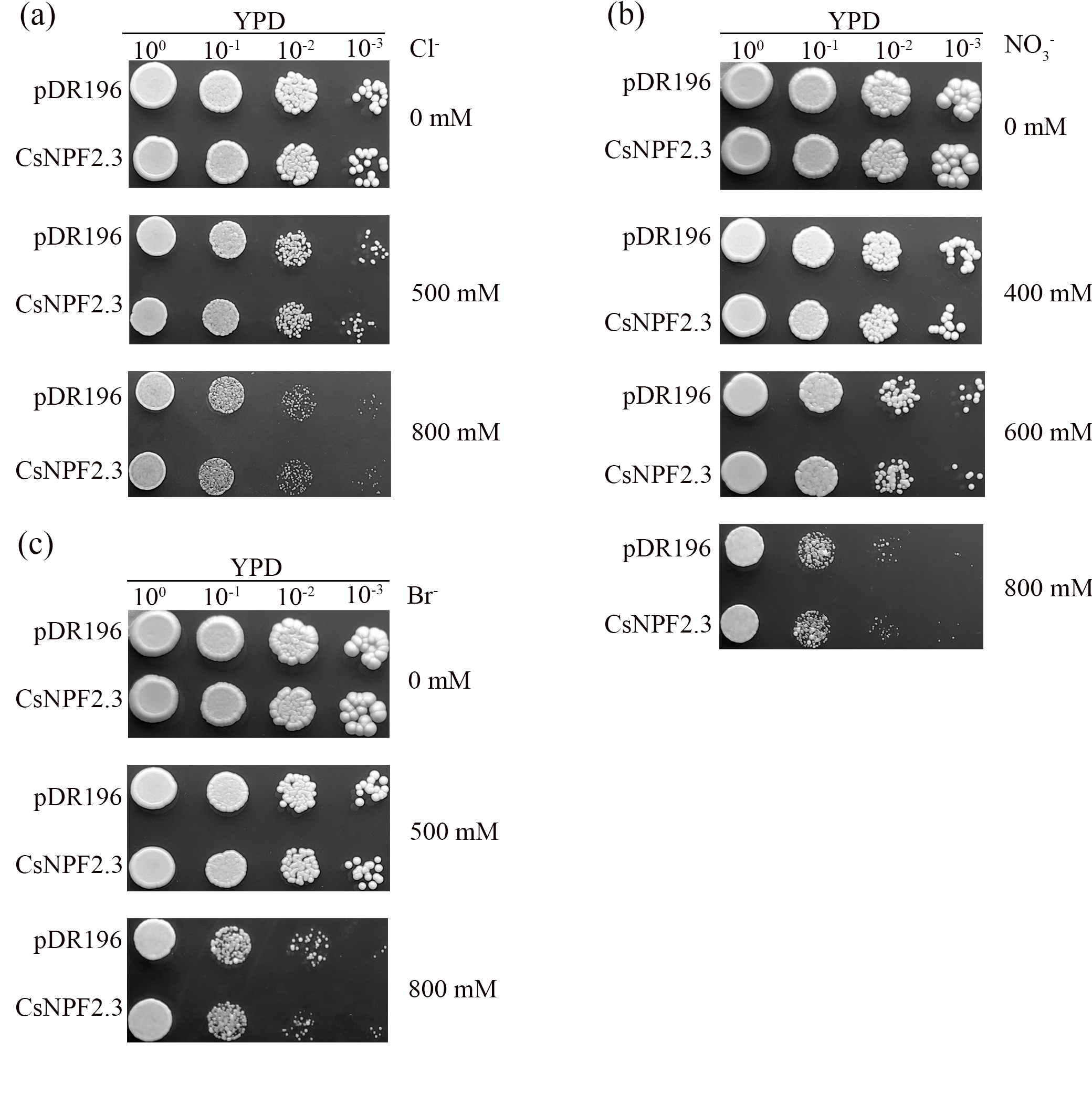


Fig. S3. Transport activities of CsNPF2.3 for NO_3_^-^ , Cl^-^ and Br^-^ in yeast. A, Cl^-^ transport activity of CsNPF2.3 in yeast for 2 d. B, NO_3_^-^ transport activity of CsNPF2.3 in yeast for 4 d. C, Br^-^ transport activity of CsNPF2.3 in yeast for 4 d.
