# Supplementary material for: Root-specific expression of CsNPF2.3 is involved in modulating fluoride accumulation in tea plant (Camellia sinensis)
Source: Hortic Res. 2025 Mar 3;12(6):uhaf072. doi: 10.1093/hr/uhaf072 (PMC12038894; doi:10.1093/hr/uhaf072)
Supplement: Web_Material_uhaf072 [file web_material_uhaf072.zip › Supplementary Table S1.docx]

Table S1 Primers required in this study

| Primer name | Primer sequence (5’ to 3’) | Annotation |
| --- | --- | --- |
| *CsNPF2.3-F* | ATGGTTGCCGGCTCACTC | Cloning of the CsNPF2.3 ORF |
| *CsNPF2.3-R* | TTACTTATCATCAGAACTAGGATTCTCA |  |
| *pDR196-CsNPF2.3-F* | ccccagcctcgactagtATGGTTGCCGGCTCACTC | Heterologous expression in yeast |
| *pDR196-CsNPF2.3-R* | agcttgatatcgaattcTTACTTATCATCAGAACTAGGATTCTCA |  |
| *CsNPF2.3-GFP-F* | aggacagcccagatcactagtATGGTTGCCGGCTC | Generation of CsNPF2.3 overexpression lines in tea plant, Subcellular localization, |
| *CsNPF2.3-GFP-F* | gcccttgctcaccatggatccCTTATCATCAGAACTAGGATTC |  |
| *CsNPF2.3-qF* | GCGACACTCGACTCTCTGAG | qRT-PCR |
| CsNPF2.3-qR | GCCGAACCCCAATCCAAAAC |  |
| *CsGADPH-qF* | TTGGCATCGTTGAAGGGTCT | Internal reference gene in C. sinensis for qRT-PCR |
| *CsGADPH-qR* | CAGTGGGAACACGGAAAGC |  |
| *situ-CsNPF2.3-F* | CCCATCCTCGGAGCAATCG | *In situ* PCR analysis |
| *situ- CsNPF2.3-R* | GGTGAACCGTGTACCTGCG |  |
