# Supplementary material for: Root-specific expression of CsNPF2.3 is involved in modulating fluoride accumulation in tea plant (Camellia sinensis)
Source: Hortic Res. 2025 Mar 3;12(6):uhaf072. doi: 10.1093/hr/uhaf072 (PMC12038894; doi:10.1093/hr/uhaf072)
Supplement: Web_Material_uhaf072 [file web_material_uhaf072.zip › Supplementary Table S2.docx]

Table S2 DEGs between F and Se + F

| Gene ID | FDR | Log_2_ Ratio ((Se + F)/F) | Up/Down ((Se + F)/F) |
| --- | --- | --- | --- |
| TEA031957.1 | 1.44867E-06 | -1.393100326 | down |
| TEA033833.1 | 1.58211E-05 | 3.13283443 | up |
| TEA007060.1 | 2.52557E-05 | 2.158234704 | up |
| TEA028550.1 | 0.000145851 | -2.335064769 | down |
| TEA019808.1 | 0.000367505 | 1.61876544 | up |
| TEA024019.1 | 0.000681804 | 0.992281397 | up |
| TEA005583.1 | 0.000949708 | 0.887558931 | up |
| TEA001993.1 | 0.00103681 | 2.013787485 | up |
| TEA004881.1 | 0.001276851 | -3.542223146 | down |
| TEA005580.1 | 0.001560409 | 0.776331117 | up |
| TEA020860.1 | 0.007118283 | -1.553431874 | down |
| TEA026555.1 | 0.009927652 | 1.925353211 | up |
| TEA021139.1 | 0.01025902 | 0.669493026 | up |
| TEA000295.1 | 0.026834164 | -0.754955599 | down |
| TEA022504.1 | 0.0446317 | -1.491650001 | down |
