# Supplementary material for: Root-specific expression of CsNPF2.3 is involved in modulating fluoride accumulation in tea plant (Camellia sinensis)
Source: Hortic Res. 2025 Mar 3;12(6):uhaf072. doi: 10.1093/hr/uhaf072 (PMC12038894; doi:10.1093/hr/uhaf072)
Supplement: Web_Material_uhaf072 [file web_material_uhaf072.zip › Supplementary Table S3.docx]

Table S3 Correlation coefficient and P-value between the expression level of DEGs encoding nitrate transporter and leaf F content and TF of F

| Gene name | F content in leaves | | Translocation factor | |
| --- | --- | --- | --- | --- |
|  | Correlation | P-value | Correlation | P-value |
| TEA031957.1 | 0.838070646 | 0.037208689 | 0.969838897 | 0.00135082 |
| TEA033833.1 | -0.622046253 | 0.187278389 | -0.773737104 | 0.071000594 |
| TEA007060.1 | -0.735217554 | 0.095882701 | -0.849800387 | 0.03214564 |
| TEA028550.1 | 0.580414127 | 0.227143927 | 0.753256175 | 0.08381258 |
| TEA019808.1 | -0.628278024 | 0.181584085 | -0.780006762 | 0.067272028 |
| TEA024019.1 | -0.761305734 | 0.078662632 | -0.83195597 | 0.039985514 |
| TEA005583.1 | -0.752412792 | 0.084360661 | -0.874360676 | 0.022686236 |
| TEA001993.1 | -0.723906136 | 0.103818716 | -0.825541384 | 0.042998819 |
| TEA004881.1 | 0.245941851 | 0.638525415 | 0.457315087 | 0.361848143 |
| TEA005580.1 | -0.693409085 | 0.126587519 | -0.828550279 | 0.041572627 |
| TEA020860.1 | 0.976662797 | 0.000810583 | 0.920456675 | 0.00923907 |
| TEA026555.1 | -0.534730361 | 0.274353939 | -0.714026367 | 0.110977785 |
| TEA021139.1 | -0.719761734 | 0.106796184 | -0.882454274 | 0.019913432 |
| TEA000295.1 | 0.871057405 | 0.023867377 | 0.958243111 | 0.002579052 |
| TEA022504.1 | 0.234400127 | 0.654839182 | 0.491464636 | 0.322156613 |
