# Supplementary figures and images for: Root-specific expression of CsNPF2.3 is involved in modulating fluoride accumulation in tea plant (Camellia sinensis)
Source: Hortic Res. 2025 Mar 3;12(6):uhaf072. doi: 10.1093/hr/uhaf072 (PMC12038894; doi:10.1093/hr/uhaf072)

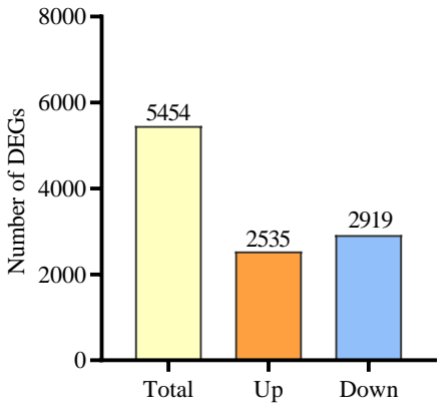

Supplement: Web_Material_uhaf072 [file web_material_uhaf072.zip › Figure S1.pdf]

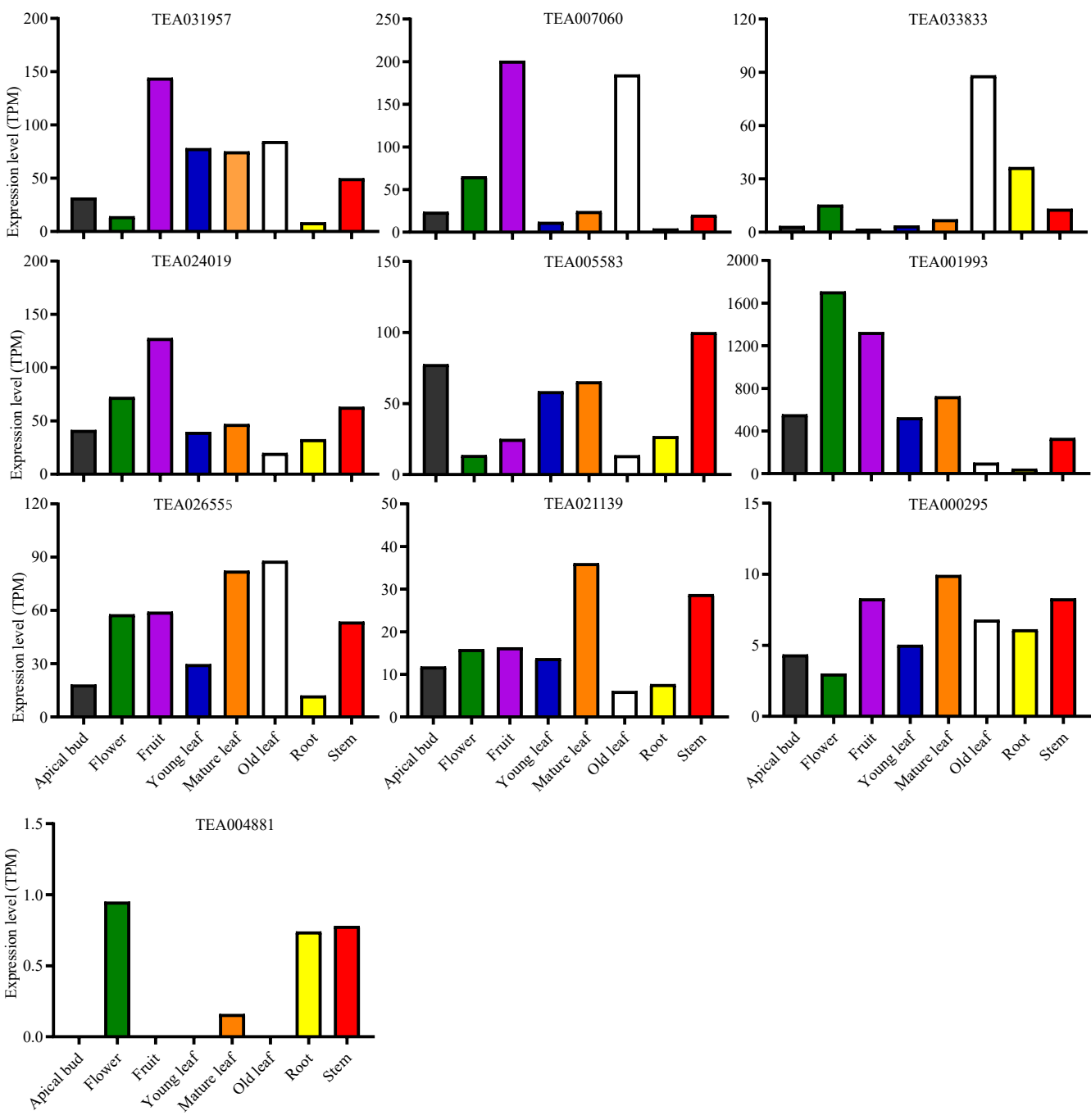

Supplement: Web_Material_uhaf072 [file web_material_uhaf072.zip › Figure S2.pdf]

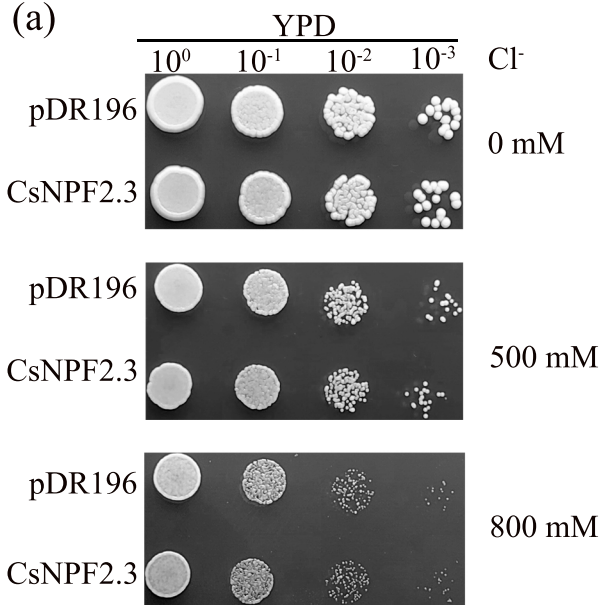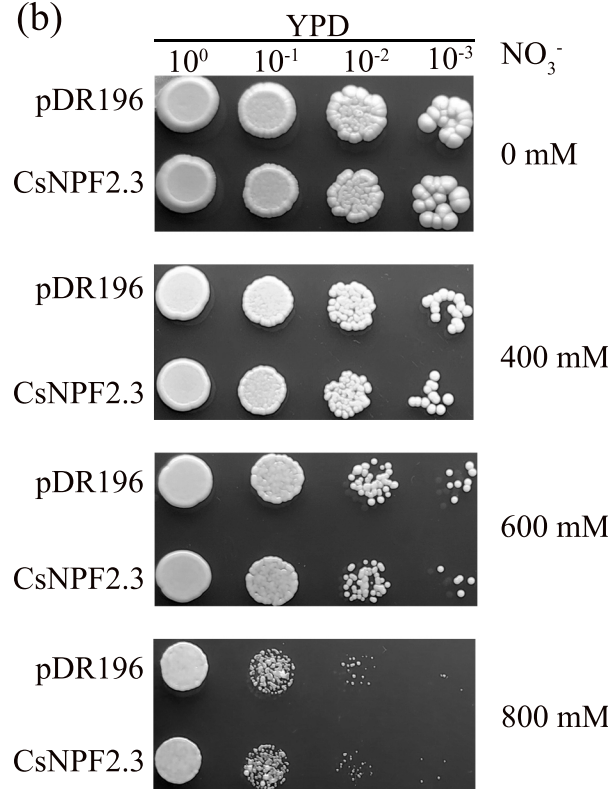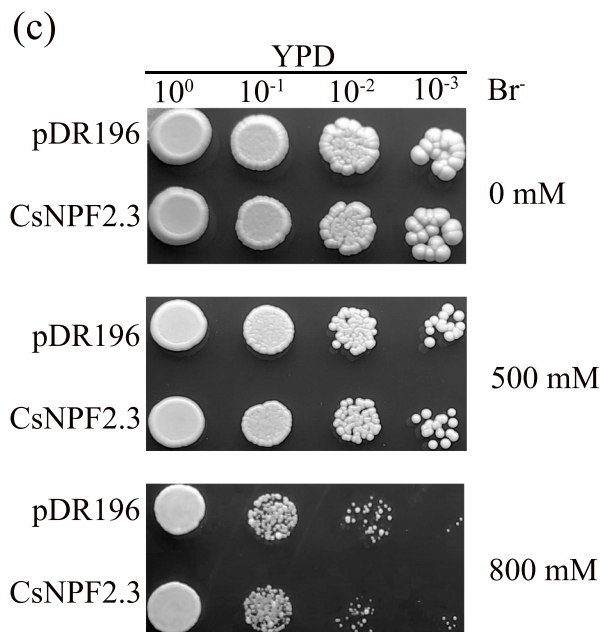

Supplement: Web_Material_uhaf072 [file web_material_uhaf072.zip › Figure S3.pdf]
